# The experience of professional identity formation by palliative care practitioners: an exploratory qualitative study by the Singapore Hospice Council

**DOI:** 10.3389/frhs.2026.1771460

**Published:** 2026-04-01

**Authors:** Eng Koon Ong, Candice Tan, Andy Sim, Min Chiam, April Thant Aung, Arista Tan, Vanessa Ho, Bee Hia Sim

**Affiliations:** 1 Homecare, Assisi Hospice Singapore, Singapore; 2 Division of Cancer Education, National Cancer Centre Singapore, Singapore; 3 Duke-NUS Graduate Medical School, Singapore; 4 Medical Humanities, SingHealth-Duke NUS, Singapore; 5 Singapore Hospice Council, Singapore; 6 Division of Supportive and Palliative Care, National Cancer Centre Singapore, Singapore; 7 Care and Counseling, Tan Tock Seng Hospital, Singapore; 8 Department of Medical Social Services, Singapore General Hospital, Singapore

**Keywords:** national strategy, palliative medicine, professional identity formation, staff support, wellbeing

## Abstract

**Background:**

Professional identity formation (PIF), the process through which professionals internalise and reconcile new experiences with a pre-existing identity can lead to better patient care outcomes and staff fulfilment. However, how palliative care practitioners (PCPs) experience PIF is currently unclear. To support services as they expand capacity to cope with increasing needs, this study addresses the question, How is professional identity formation experienced by palliative care providers in Singapore?”

**Methods:**

This exploratory qualitative study investigates how PIF is experienced by PCPs through a Relativist ontological position and a Constructivist epistemological lens. A total of seven focus group discussions involving 35 representatives from 19 member organisations of the Singapore Hospice Council (SHC) were conducted between August and September 2024. Each session lasted approximately 1–1.5 h. The data was audio-recorded, anonymised, transcribed, and analysed thematically.

**Results:**

Three themes and 7 subthemes are identified. The three themes are: prevailing professional identity of PCPs, dilemma between professional self-sufficiency and interprofessional collaboration, and expectations of leadership.

**Conclusion:**

This study elicited a self-reported, multi-faceted description of professional identity by local PCPs. PCPs’ expectations of their professional competence, together with insights about how interprofessional collaboration is practiced and expectations of organisational leadership reinforce the need to adopt a multi-pronged approach towards staff support. Such an approach should include developing PCPs’ competence across both biomedical and psychosocial domains, foster IPC practices that translate to staff support, and alignment of organisational goals with service realities. Future research should evaluate the implementation and effectiveness of such approaches.

## Introduction

Palliative care (PC) may be defined as an approach that ensures holistic care for patients with life-limiting conditions and their caregivers across all domains of physical, psychological, and spiritual care (1). The rising demands of patients who require palliative care (PC) support has made the development of palliative care services a global imperative (2). Expanding the availability of expertise, resources, and access to care will allow patients to access crucial medicines, experience better symptom relief and psycho-emotional support, and fulfil their care preferences (2).

The European Association for Palliative Care (EPAC), the American Academy of Hospice and Palliative Medicine (AAHPM), the Canadian Hospice Palliative Care Association (CHPCA) and the Asia-Pacific Hospice Palliative Care Network (AHPCN) are some examples of organisations globally that aspire to increase awareness about palliative care (PC), improve quality of care, ensure equity of care, and influence policies to support palliative care systems. In view of these aspirations, how professional identity formation (PIF) is experienced by palliative care providers (PCPs) warrants further investigation (3).

Professional identify formation (PIF) may be described as the process whereby healthcare workers reconcile pre-existing personal values with professional expectations and aspirations (4). These facets of professional identity are shaped by interprofessional relationships, the working and learning environment, organisational structure, and patient encounters (5). Illuminating the impact of prevailing culture and values, relationships, reflective practice and the philosophy of palliative care, the concept of PIF allows a holistic understanding on how PCPs may be supported as they heed the call to address global and local palliative care needs in the aspects of capacity expansion, evolving professional roles, and changes to national policy changes (1, 6, 7). In healthcare, PIF that is supported within a nurturing environment allows professionals to provide better care, achieve self-actualisation, and reduce burnout (8). Conversely, conflicts and tension between pre-existing values and beliefs and what is experienced can lead to moral distress, burnout, and attrition (9).

In PC, nurturing PIF is especially important due to the unique pressures of managing complex physical and psychosocial needs of patients, the inherent emotional toll of regularly encountering death and grief (10), and the tension between service demands and systemic limitations in manpower and other resources (11, 12). These challenges are further compounded by ethical dilemmas encountered in PC, including withdrawal of hydration and nutrition at end of life (13, 14), collusion of medical information (15), and requests for hastened death and euthanasia (16).

In Singapore, the impetus for a system-wide examination of the needs of PCPs has been highlighted in the past decade as the population ages. Singapore is expected to attain “super-aged” status in 2026, with one quarter of its citizens aged 65 and above (6). To ensure sustainability of healthcare resources, the Ministry of Health (MOH) Singapore has implemented initiatives that focus on preventive care, social determinants of health and health literacy and awareness (7). In view of the above, palliative care has gained increased attention in recent years for its role in supporting Singapore's evolving healthcare landscape. Specifically, the wellbeing of PCPs has come under the spotlight.

In Singapore, PC is provided in acute care hospitals, and in the community through home care, daycare, and inpatient hospice care (17). PC teams in these settings receive referrals from the primary physicians of patients based on their needs and preferences. This triad of PC care settings ensures that patients receive care across a spectrum of intensity and fulfil their preferences for places of care and end of life (17). It also allows patients to transition across settings smoothly based on evolving needs. PCPs work in interdisciplinary teams which include PC physicians, nurses, medical social workers, physical therapists, creative arts therapists, and pastoral care counsellors (16, 18).

With PC's holistic approach towards care, it is well-positioned to support the care and management of patients in an aging population. Early access to PC has been demonstrated to reduce inappropriate healthcare utilisation, reduce healthcare costs (19), and improve patient satisfaction (20). In 2023, the number of people receiving community palliative care services in Singapore increased by 30%, a trend that reflects an exponential need to develop PC services to meet rising demands (7). However, Singapore's National Strategy for Palliative Care (NSPC) that was updated in 2023 to guide the development of PC services (21) does not provide guidance on PIF despite its potential in sustaining PCPs in achieving national goals. A literature review of global data also suggests a gap in how the PIF of PCPs is understood. The variability and needs of different PCPs across settings further highlight an urgent need to understand how PIF is experienced by PCPs. For example, home hospice PC teams have been noted to be at the highest risk of burnout due to limited resources, intensity of practice, and lack of manpower (3, 5, 22–24). Junior doctors and senior nurses providing PC in acute hospitals struggle with interpersonal relationships, balancing service and training requirements, and the hidden curriculum (24, 25). There is also conflicting data on whether palliative care's focus on an interdisciplinary and person-centred care translates to better support for PIF. Some researchers have argued that such an approach may promote collegiality, teamwork, and positive role-modelling (26–28), but others have suggested that tensions between service demands vs. aspirational philosophy of practice may lead to a sense of cynicism, helplessness and discontent (29).

To address the above gaps in knowledge, the Singapore Hospice Council (SHC), with the support of MOH Singapore, embarked on this study that included all local palliative care services. It is hoped that the insights from this study will also provide guidance and inspire other global networks to embark on similar studies and establish systematic guidelines to support PIF in PCP.

The SHC was first established in 1995 as a registered charity and is recognised as an Institution of Public Character (30). In addition to pursuing its vision of supporting quality palliative care for the local population, SHC is also an umbrella body that represents all organisations providing palliative care services in Singapore. At the time of this study, SHC had 25 member organisations spread across acute restructured hospitals, hospices, and academic institutions. These included 10 palliative care services in acute hospitals, 13 organisations that provide inpatient hospice care, home care, or daycare, and 2 academic and research centres.

SHC's mission to be the collective voice of the palliative care community and to advance the development of the discipline has led to regular engagement with the public through roadshows, the curation and sharing of educational resources amongst PCPs, and collaboration with regulatory bodies on policymaking and standards-setting. This puts SHC in a unique position to play an important leadership and collaborative role in spearheading research and programmes that promote the PIF of PCPs in Singapore.

### Research question

This is an exploratory qualitative study that employs focus group discussions with representatives from the member organisations of the Singapore Hospice Council. The study addresses the research question: How is professional identity formation experienced by palliative care providers in Singapore?

### Theoretical framework

A review of the existing literature suggests that the concept of professional identity formation (PIF) is a sociocultural construct that is heavily influenced by individual interpretations of contextual, sociocultural, and systemic experiences (23, 24). Such concepts demand consideration of the palliative care provider's belief systems, narratives, contextual considerations; and developing competencies (3, 5, 31). The interplay between multiple individual and environmental factors that contribute to PIF suggests that there is no single “right”, or objective experience of PIF and an exploratory approach is crucial (32). As a result, a Relativist ontological position and a Constructivist epistemological lens is adopted to investigate PCPs’ PIF given that knowledge and understanding of these features are based on contextual factors. This individually- and systemically-informed understanding of socio-culturally influenced and evolving concepts of knowledge, insights, meaning making and reflections also relegates the possibility of positivist and postpositivist perspectives (33–35). This places a constructivist approach and relativist lens at the heart of the methodological approach of this study.

This study is guided by Cruess’ framework that describes the fluctuating process of PIF where professionals first enter the profession with a pre-existing personal identity shaped by personal experience and values (36). Through a process of socialisation which involves negotiation and acceptance or rejection of new experiences and interactions within a community of practice, a new professional identity is developed (37). This process of socialisation is shaped by role models, clinical and non-clinical experiences, the learning environment and healthcare system, and treatment from patients and colleagues. These factors thus provided the structure through which the research question of this study could be investigated.

## Methododoly

### Study design

This is an exploratory qualitative study that adopts a Relativist ontological position and a Constructivist epistemological lens, acknowledging the need for crucial bidirectional interactions to delve into individual experiences, insights, reflections, attitudes, and beliefs, the study design employed qualitative methods in addressing the research question (38). In this study, professional identify formation (PIF) is understood to be the process whereby healthcare workers reconcile pre-existing personal values with professional expectations and aspirations (4).

### Context and environment of practice

Although all palliative care providers (PCPs) adopt an interdisciplinary approach towards holistic care, the 25 member organisations under the umbrella of the Singapore Hospice Council vary in terms of size, clinical practices, and resources. For the 10 organisations situated within the acute hospitals, the PC teams function as specialist medical teams that provide consult for patients with life-limiting conditions admitted to the acute hospitals. Sharing a common leadership and management structures as other hospital departments, PCPs have access to common infrastructural resources, manpower, medications, and equipment.

In contrast, the 13 community palliative care organisations that provide home care, daycare, or inpatient hospice care services are independent voluntary welfare organisations with their independent boards of governance and clinical teams. Patients are referred to these services by their primary physicians when acute care is no longer needed. The community palliative care organisations are supported by public donations and government subvention. These organisations may also have less access to manpower and other resources, including access to programs for staff wellbeing (17).

### Researcher reflexivity

The study team comprises of a senior palliative care specialist working in a home hospice setting, two senior palliative care medical social workers working in acute hospitals, two senior research assistants experienced in qualitative research, and three senior administrative staff from the Singapore Hospice Council. All members of the study team have experience in education and research programs involving PCPs. Taking a reflexive approach, the study team recognises that this study may be considered as insider research. Insider research may be defined as research that is conducted within a social group, organization, or culture of which the researcher is also a member (39). As fellow members of the palliative care (PC) community, the members of the study team share similar experiences and characteristics as the participants of the study. Whilst this helps in the overall understanding of the subsequent data collected, the study team is also mindful of the inadvertent biases and perspectives that may be injected during data collection and analysis. To avoid this, the inclusion of two senior research assistants outside of the PC community in data collection and analysis ensures that an objective and curious lens is employed. The risk of biases is also avoided as we adhere to established qualitative research methods which are described in further details below.

### Participants recruitment

Email invitations were sent to all 25 SHC member organisations between June and July 2024. Each organisation was invited to nominate two staff members to participate in focus group discussions (FGDs) on staff wellbeing. The inclusion criteria required participants to have at least one year of employment at the organisation, involvement and expressed interest in staff wellbeing, and availability to attend a single FGD not exceeding 90 min.

No restrictions were placed on profession and seniority, and administrative and management staff were eligible to participate. Staff employed on a temporary basis or had little involvement in staff wellbeing were excluded.

The inclusion and exclusion criteria were listed based on several factors. Firstly, the participants had to be both interested in staff support and aware of current or future initiatives to be able to contribute meaningfully to the FGDs. It was also recognised that staff members of each organisation who were designated to support staff wellbeing may not always be clinicians, thus administrative and management staff were not excluded. Finally, exclusion of non-clinicians may reduce the number of potential individuals available for this study in smaller organisations, thus affecting recruitment.

The Participant Information Sheet (PIS) and consent forms were attached electronically in the invitation emails. The PIS provided the background information of the study, including the research question, data collection methods, and details about the principal investigator. The identified staff members then provided written consent by replying to the invitation emails with their contact details.

### Data collection

The participants who provided written consent were then contacted by an independent research assistant (RA) to arrange for the focus group discussions (FGDs). Before each FGD, participants completed an online questionnaire covering both individual and institutional demographics. These included age, gender, profession, setting of practice (hospital vs. community) and type of service provided (referral service vs. primary team care). The questionnaire also gathered information on organisational size (staff and patient numbers), the presence of a 24 h on call service, and participants’ working hours. These factors were selected for their potential impact on PIF (23, 24, 31, 32).

FGDs were chosen as the method of data collection as participant interactions would encourage the emergence of new perspectives through the interactive sharing of common experiences (40). An interview guide was designed for the purpose of the study and consisted of both prompting and probing questions. The questions were created based on a literature review on the topic and the theoretical framework described above. The interview followed the three domains of workplace culture, the impact of caring for palliative care patients and the effects of organisational and systemic policies on staff wellbeing. The semi-structured nature of the interview guide with pre-determined questions that were open-ended allowed focused yet flexible discussions (33). This was important to ensure a guided yet non-restrictive collection of data in view of the diverse perspectives and experiences of different participants.

Participants were assigned into groups of three to six and each FGD was limited to no more than 90 min to minimise fatigue. FGDs were conducted in-person by an independent research assistant at the premises of the SHC and were audio-recorded, anonymised and transcribed for analysis. Each participant was assigned a unique identifier to ensure anonymity.

### Data analysis

Thematic analysis of the transcripts was performed using Braun and Clarke's method of reflexive analysis (41, 42). Members of the study team engaged in individual reflections and group discussions to reach consensus on identified themes. Thematic analysis is a qualitative research method that “recognises, investigates and categorises patterns within data” (41) and allows data to be further interpreted so that new insights into different perspectives from the participants may be elicited. A reflexive approach enabled data analysis to be flexible and iterative in nature (42, 43). As the members of the study team started the coding process, evolving interpretation of the data correspondingly consolidated conceptualisation of the themes elicited.

The process of data analysis involved multiple steps and started with the creation of familiarisation notes using Microsoft Excel (2018). Reading of the transcripts was done with an open mind. In an analytic manner, points of interest were noted. This led to the generation of initial codes and collation of points that were relevant to the research question, allowing members of the study team to reflect and make sense of what was shared by the participants. Initial themes were then created, allowing the consolidation of common patterns within the codes. Repeated reflections on the codes and review of the themes led to the creation of an early thematic map, which was subsequently finalised after consideration of descriptive and concise names.

### Rigor of data collection and analysis

The research assistant (RA) was trained by the study team members for the conduct of the FGDs. The RA had a Master's degree and possess significant experience in conducting similar qualitative studies in healthcare research. The training supplemented her existing competencies by providing further information on local palliative care services, the role of the SHC, and the overall goal of the study to support local PCPs effectively and efficiently.

In addition, the RA was trained in group facilitation skills that included the ability to recognise both verbal and non-verbal cues and manage intra-group interactions, conduct open-ended questioning, set ground rules at the beginning of each FGD which emphasize respect and confidentiality, respond in a non-judgemental manner, and to provide basic psychological “first-aid” to participants if necessary. Practice interviews were also conducted for the RA to gain familiarity with the interview guide and receive feedback.

As a trial employment of the interview questions was not possible with the study's participants beforehand, three members of the study team participated in a role-play scenarios with the RA as practice and to address any concerns or questions by the RA in the use of the interview guide. The two members of the study team took on roles in three different settings to simulate the conduct of the actual FGDs.

To ensure an unbiased and credible data collection process, the employ of the RA as an independent party in the conduct of the FGDs promoted psychological safety for the participants. All other members of the study team would only receive anonymised data for the purpose of analysis after the FGDs were completed. In addition, before the start of each FGD, the RA emphasized to the participants her independent role, the anonymisation of data, and the assurance that their sharing will not impact their appraisal or work processes. The participants were also reminded that they could leave the study at any point of time if they felt uncomfortable without the need to provide a reason to do so.

To ensure dependability and an objective approach towards data analysis, a reflexive approach that includes individual self-reflection during the initial familiarisation of each transcript was strictly adhered to. This reflexivity was further applied through peer-critique during code and theme formation to ensure coherence and triangulation of analysis to avoid biases. Finally, an additional and fresh lens was applied by the two non-palliative care research team members (MC, ATA) before finalisation of codes and themes.

## Results

Seven FGDs were conducted between August and September 2024, involving 35 participants from 19 SHC member organisations. Each FGD lasted between 68 and 92 min.

Participant demographics are shown in Table 1. Most of the participants were nurses, physicians, and medical social workers. Acute hospitals were represented by 18 participants (approximately 50% of participants) and had more staff than community organisations, although that did not correspond to the size of the patient population under their care. Most of the participants shared that their staff worked between 40 and 55 h per week, and 29 participants (approximately 81%) reported an after-hours on-call system in their institutions.

**Table 1:** Participant demographics.

S/N	Gender	Age	Profession	Setting of practice	Size of organisation: number of staff	Size of organisation: number of patients	Presence of 24 h on call service	Working hours per week
PCP 1	Female	43	Nurse	Acute hospital	5	5	No	164
PCP 2	Female	37	Consultant doctor	Acute hospital	6	1,200	Yes	252
PCP 3	Female	29	Nurse	Home care/inpatient hospice/hospice daycare	8	130	Yes	45
PCP 4	Female	34	Nurse	Home care/inpatient hospice/hospice daycare	[Response omitted for anonymity]	[Response omitted for anonymity]	Yes	44
PCP 5	Female	49	Nurse	Acute hospital	∼500	243	Yes	42
PCP 6	Female	60	Chaplain	Acute hospital	20+	16+	Yes	42
PCP 7	Female	40	Medical social worker	Acute hospital			No	29
PCP 8	Female	41	Nurse	Acute hospital	40	200	Yes	45
PCP 9	Female	38	Nurse	home hospice	187	∼3,800 patients and their families	Yes	425
PCP 10	Female	55	Nurse	Home Care/Inpatient Hospice/Hospice Daycare	10	160	Yes	40
PCP 11	Female	30	Nurse	Home Care/Inpatient Hospice/Hospice Daycare	4	98	Yes	40
PCP 12	Female	37	Consultant doctor	Acute hospital	3	On average, 20 patients in the team	No	55
PCP 13	Male	28	Nurse	Acute hospital	6	15–20	No	40
PCP 15	Female	49	Nurse	Acute hospital	32	20	Yes	40
PCP 16	Female	39	Nurse	Home care/inpatient hospice/hospice daycare	Unsure	400–500 per year	Yes	44
PCP 17	Female	43	Medical social worker	Home care/inpatient hospice/hospice daycare	Unsure	85	Yes	42
PCP18	Female	37	Nurse	Home Care/Inpatient Hospice/Hospice Daycare	40	600	Yes	42.5
PCP 19	Female	36	Registrar physician	Home care/inpatient hospice/hospice daycare	30 (doctors)	34	Yes	40
PCP 20	Female	52	Medical social worker	Home care/inpatient hospice/hospice daycare	[not provided]	[not provided]	Yes	40
PCP 21	Male	35	senior resident physician	home care/inpatient hospice/hospice daycare	55	195	Yes	82
PCP 22	Female	36	Dietitian	Acute hospital	Not sure	Not sure	No	42
PCP 23	Female	49	Nurse	Home Care/Inpatient Hospice/Hospice Daycare	6	100	Yes	42
PCP 24	Male	38	Medical social worker	Home care/inpatient hospice/hospice daycare	205	3,558	Yes	45
PCP 25	Prefer not to say	28	Medical social worker	Home care/inpatient hospice/hospice daycare	Over 100	Over 400	Yes	40
PCP 26	Female	42	Nurse	Acute hospital	Close to 5,000 (nurses)	∼3,000	Yes	42
PCP 27	Female	38	Consultant doctor	Acute hospital	10,000	800 inpatient beds	Yes	45
PCP 28	female	42	nurse	home care/inpatient hospice/hospice daycare	23	20	Yes	45
PCP 29	Female	36	Consultant doctor	Acute hospital	10	6–10 under primary, 2–10 under consult	Yes	48–50
PCP 30	Female	41	Medical social worker	Home care/inpatient hospice/hospice daycare	[not provided]	[not provided]	Yes	[not provided]
PCP 31	Male	40	Consultant doctor	Acute hospital	4,000	800	Yes	50
PCP 32	Female	36	Nurse	Acute hospital	7	15–22	No	40
PCP 33	female	45	Nurse	acute hospital	[Not provided]	[Not provided]	Yes	8
PCP34	Female	55	Nurse	Hospital	50	Missing data	Yes	44
PCP35	Female	34	Doctor	Hospital	50	Missing data	Yes	Missing data
PCP 36	Female	60	Head (Human Resources)	Home Care/Inpatient Hospice/Hospice Daycare	215	240 (includes both hospice & palliative)	Yes	44

Analysis of the anonymised transcripts identified three main themes with accompanying subthemes. The main themes were 1) prevailing professional identity of PCPs, 2) dilemma between professional self-sufficiency and interprofessional collaboration, and 3) expectations of leadership.

### Prevailing professional identity of PCPs

Analysis of the data suggests that PCPs anchored PCPs’ professional identity around professional competence. This theme encompasses 3 subthemes, namely how professional competence is perceived, main motivations of PCPs to enculturate to PC's unique philosophy of care, and why PCPs resist roles that are felt to be administrative in nature.

#### Defining aspects of professional identity

Professional competence was first described as the ability to address PC issues faced by patients across all physical, psychosocial, and spiritual domains.

PCP1(FGD1) shared how

“for the symptoms actually we do have to address to that and make sure the patient is well settled before we move onto another. And the other one is difficult family conversation (to address psychosocial distress) that we have that we can be there…”

However, the ability to cope with unexpected patient complexities and prognositcation was also needed.

PCP29 (FGD4) shared how it may be

“ …seasonal variation… (you may) manage a ward that has many patients with complex needs… for that month naturally your stress level will be higher. If you have difficult family for that month naturally your stress level will be higher.”

PCP15 (FGD3) recalled the impact on staff when patients’ prognosis changed and they deteriorated or died unexpectedly:

“…two colleagues developed… insomnia… one colleague… resigned because she couldn't handle patients who [were] dying… our (nursing) sister just let her go because it affect[ed] her mentally.”

Finally, staff also had to learn to cope with uncertainty about manpower and team composition, as described by PCP14 (FGD7):

“…[teams] keep changing. If you get people you can work well with… then it's easier. But sometimes if you have a difficult colleague then that will affect the quality [of care].”

#### Motivations to enculturate to PC's unique philosophy of care

Despite the difficulties faced by PCPs, PCPs were committed to internalise PC's holistic approach to patient care into their professional identity. Extrinsic motivations include a supportive working environment where hierarchical conflicts were absent. PCP 9 (FGD2) shared how

“…even our CEO (Chief Executive Officer) would say, ‘oh don't tell me nice things (only) …if not we won't know where to improve or where to work on.’ She's very open so she will come down personally… to meet the staff and do all this.”

Intrinsically, PCPs like PCP13 (FGD3) found meaning and professional satisfaction in their work when their efforts were acknowledged by patients:

“…it's the simple appreciation. Even if my patient says, ‘Thank you for whatever you have done.’… it just like gives you a sense of purpose and satisfaction…like whatever you are doing is the right thing. It pushes you or motivates you to keep doing what you are doing.”

However, these motivations sometimes reduced PCPs’ wellbeing when self-sacrifice came to be perceived as both necessary and desirable:

“[There is] no such thing as, ‘I [am] off duty. You don't disturb me.’ No, you can't say [that]. Our promise is 24/7 support… the person who [doesn't] know the patient well may make wrong decisions. That's why some… even [when] they are on holiday, they will still say, ‘never mind, call me.’” (PCP10, FGD2)

#### Difficulty in fulfilling administrative roles

With professional identity largely defined by competence associated with patient care, it was unsurprising that participants felt more comfortable in their clinical roles, resisting recruitment into other areas such as research, health services, or education. For example, PCP26 (FGD5) shared that:

“It's really not sustainable…they gave me some admin(istrative) hours… because everybody is talking about outcome. Everybody is talking about KPI (key performance indicator). But…’what's the outcome? The outcome is really the patient care.” But they must say that, “APN (advanced practice nurse) must be driven! And must show an (alternative) outcome…’ So that's a stressor. It's really a stress actually.”

The possibility of expanding one’s portfolio was also challenging even if one was willing to, as PCP 22 (FGD4) explained:

“…the manpower … is a bit low in our setting… actually we have a lot of cases… to cover. There's a lot of things they want me to have in my setting and I can't due to limitation (of time). Though we do have other projects to help to cover that but I feel that… even though I want to do good but I can't.”

PCP27 (FGD5) similarly shared that

“…the stress from the clinical [side of] work is mostly manageable but… the administrative burden… Everybody gets [into] this KPI (key performance indicator) meeting frenzy… Projects are popping up all over the place but the people doing the projects are the same few…So then um you (are) just being pushed on many fronts…the palliative care who are this small community but then you (are) pulled in every single workgroup”

### Dilemma between professional self-sufficiency and interprofessional collaboration

In describing how individual PCPs perform their clinical duties, traditional ideals of interprofessional collaboration (IPC) were challenged. For example, IPC focuses on the interdependent nature of different healthcare professions who work together to achieve common goals in patient care and inter-professional support. IPC is thus guided by effective communication, and mutual respect and trust. However, other commonly cited tenets of IPC, such as role clarification and shared decision-making, were overshadowed by the need for self-sufficiency.

#### Blurring of inter-professional roles

Despite the presence of interdisciplinary teams, inter-professional roles often overlap and had to be fulfilled by single individuals. PCP 18 (FGD 4) recalled that this affected wellbeing:

“I think the stress level is really very high especially in the nature of the work we do. As a home care nurse um I’m the case manager then sometimes when I step into the patient's house you know we are wearing different hats. So sometimes the family expect you to be a doctor, to be a social worker, physiotherapist, occupational therapist, speech therapist, everything yah.”

#### Peer support as an intra-professional duty

This expectation of being self-sufficient further translated into how peer support was provided intra-professionally. For example, PCP5 (FGD1) took it upon herself to

“…step up [to] counsel staff members and… encourage [other] staff to pull this staff up. [To] talk to each other… to understand why. Maybe certain staff [are] undergoing certain problem[s]… Don't just jump to conclusion[s].”

PCP33 (FGD6) shared how she would

“…keep an eye on (new) nurses…they come from very different background[s], from different countr[ies]… The one[s] without palliative experience [are] young and from [a] different culture (than the patients)… some of them are very young and have children at the same age… so this is something that can affect the wellbeing of the nurses…sometimes they get withdrawn and… uneasiness so they need some closure so that's when you bring them to the funeral as a team”.

Conversely, formal staff support programs which were not informed by staff themselves were poorly received, as PCP3 (FGD1) explained,

“…not many… are very open to speaking one-to-one with [the counsellors] because that's someone [they] don't know, even though it's anonymous… [They’re] not comfortable enough.”

### Expectations of leadership

Finally, having described a professional identity defined by professional competence and independence, the participants expected their leaders to live up to similar standards. PC leaders who did not serve alongside their staff were perceived to be dissociated with the realities on the ground. This sometimes led to a sense of resignation and resentment which affected the wellbeing of staff.

#### PC leaders’ “right” to lead

The participants shared how PC leaders had to be seen as contributing to service needs, before staff agreed to proposed organisational goals. As one participant expressed:

“…sometimes [leadership] rush[es] here [and] there. They want to make the team better but… don't understand what I am going through. They say, ‘how come I feel like you [are] underperforming? How come after all these years, you still cannot complete your notes on time?” (PCP16, FGD4)

Similarly, adopting similar performance indicators for both PCPs and non-PCPs prompted a sense of frustration for PCP12 (FGD 2), who lamented that

“…setting KPI (key performance indicator) in a (palliative care) clinic… and compare with other (clinics) and suggest that ‘eh how come your KPI is only this number of patients but like oncologists is like this number of patients’ (when) actually the amount (of time) that you have to spend with each patient is different. The nature of- the conversation is gonna be different yeah.”

#### Sense of resignation and resentment

As staff continued to be unconvinced of their leaders’ goals and vision, a sense of resignation started to develop as a top-down approach towards policies were experienced. PCP29 (FGD6) felt that

“…where it involves hospital-wide… trying to open more wards, we may not always have a say. It causes strain and stress to those on the ground because it's a sense of… you cannot control what is to come.”

A sense of resentment led PCP 31 (FGD6) to share how

“… the new (appointment holder) wants to have uh bonuses tied to the number of patients we see both in-patient as well as out-patient… so (it) becomes a quantitative thing instead of quality…in pall(iative) care it is really the quality 3 h I spend with the patient (but) … doesn't get translated into anything and quite clearly… for me and my other… colleagues, we feel that we will surely lose out… this is the kind of messaging that they want to send… we don't feel appreciated.”

## Discussion

In answering the research question of this study, “How is professional identity formation experienced by palliative care providers in Singapore?”, analysis of the data from this study provides novel insights about the various aspects of professional identity and aspirations of PCPs.

### Prevailing professional identity of PCPs

PCPs described themselves as clinicians who are adept in addressing physical, psycho-social, and spiritual needs of patients; able to cope with the professional grief they encounter as patients deteriorate and demise; and who also manage systemic challenges such as fluctuations in manpower availability and team composition. This multifaceted expectation of PCPs’ professional identity has been similarly described in studies which focused on home hospice nurses (3), advanced practice palliative care nurses (25), and even paramedics who provide home hospice care (44). Compared to other disciplines, ideals of competence vary. For examples, professional identity for surgeons centre around competence and confidence in their surgical skills (45). The professional identity of internal medicine physicians appear to be defined by their level of medical knowledge, and ability to produce research outputs (46). Consequently, imposter syndrome—where self-perceived incompetence occurs despite objective achievements afflict surgeons and internal medicine physicians, resulting in an inability to “fit in” (46).

The challenges PCPs face also appear to be wider in scope. Besides biomedical knowledge and technical expertise, they must also learn to manage complex emotions (47), initiate teambuilding efforts, and reconcile tensions between service and aspirational needs (48). The role of training programs for both new recruits and current PCPs in encompassing all the above aspects is thus crucial when considering interventions to support PIF (3, 49, 50).

### Dilemma between professional self-sufficiency and interprofessional collaboration

The role of interprofessional collaboration (IPC) in shaping professional identity is also highlighted. IPC has traditionally been associated with the practice of PC, with clinical platforms such as interdisciplinary meetings (IDMs) and clinical rounds involving doctors, nurses, social workers, and other members of the allied health professionals a regular fixture (51). However, suggestions from existing literature that IPC may improve wellbeing through the sharing of professional struggles and processing of complex emotions were not apparent in our results (52). In fact, the need to perform their duties efficiently by being self-sufficient and independent professionals suggests that the potential of IPC in supporting wellbeing may have been neglected in PC, possibly putting PCPs at risk of working in isolation (52).

The resultant blurring of professional boundaries in fulfilling roles beyond one's profession is however not unique to PC and has been described in doctors, nurses, and students in other disciplines (53). It is unclear from our results whether the demographics of PC patients, the nature of their biomedical and psychosocial needs, and PCPs’ strong sense of duty compel them to cross professional boundaries, inadvertently affecting their wellbeing (54). Evidence suggests that individual beliefs, experience, and willingness to reflect on ethical and professional dilemmas may explain how healthcare providers decide to cross professional boundaries in practice (55). Future studies that investigate these factors can illuminate how PCPs may be better supported in this aspect.

### Expectations of leadership

The attention to the development of palliative care (PC) services is not new. Globally, there is a call to improve access to palliative care support (2). This has led to policies and processes that increase the availability of common PC drugs, audits of clinical care outcomes, development of PCPs’ training, and advancement of research (2, 42). However, the “growing pains” of the relatively young specialty challenges PC organisations and leaders to address issues such as limited manpower, education needs of PCPs, capacity for service expansion, and reduced staff wellbeing (17, 47, 48). It is unclear if current systems and leadership in PC are adequately equipped with the necessary skills to do so (56). Based on analysis of our data, we posit that attributes like authentic leadership style (56), the ability to reconcile differences in expectations between staff and leadership (57), and most importantly role-modelling professional competencies (58) are crucial. The EAPC has proposed a framework for leadership training consisting of 8 domains such as teamwork, project management, communication, and advocacy (59). However, nuances across different PC settings need to be considered so that local needs can be met (60–63).

## Limitations

This study is limited by its study design in several aspects. The variability of participant demographics poses unique challenges in how the data could be collectively analysed. Local context and experience may also differ in other countries and systems, risking lack of generalisability of the results. Lastly, triangulation of the data is not possible as insights from senior leadership and policy makers are not available, risking a possible degree of bias from ground staff and middle management.

## Conclusions

This study conducted by the Singapore Hospice Council (SHC) elicited a self-reported, multi-faceted description of professional identity by local palliative care providers (PCPs). PCPs expectations of their professional competence, together with insights about how interprofessional collaboration is practiced and expectations of organisational leadership reinforce the need to adopt a multi-pronged approach towards staff support. Such an approach should include developing PCPs’ competence across both biomedical and psychosocial domains, foster IPC practices that translate to staff support, and alignment of organisational goals with service realities. Future research should evaluate the implementation and effectiveness of such approaches.

## Data Availability

The original contributions presented in the study are included in the article/Supplementary Material, further inquiries can be directed to the corresponding author.
